# Estimation of Free Sugars in the Filipino Food Composition Table and Evaluation of Population-Level Intake

**DOI:** 10.3390/nu15061343

**Published:** 2023-03-09

**Authors:** Fabio Mainardi, Vanessa Caroline Campos, Richard Gaston Côté, Nele Kristin Silber, Roko Plestina, Imelda Angeles-Agdeppa

**Affiliations:** 1 Nestlé Institute of Health Sciences, Nestlé Research, Vers-chez-les-Blanc, 1000 Lausanne, Switzerland; 2 Department of Science and Technology, Food and Nutrition Research Institute, Gen. Santos Avenue, Bicutan, Taguig City 1631, Philippines

**Keywords:** food composition database, free sugar, missing value imputation

## Abstract

Recommendations to reduce intake of free sugars are included in some national dietary guidelines. However, as the content of free sugars is absent from most of the food composition tables, the adherence to such recommendations is hard to monitor. We developed a novel method to estimate the free sugar content in the Philippines food composition table, based on a data-driven algorithm that enabled automated annotation. We then used these estimates to analyze the free sugar intake of 66,016 Filipinos aged 4 years and over. The average free sugar consumption was 19 g/day, accounting for an average of 3% of the total caloric intake. Snacks and breakfast were the meals with the highest content of free sugars. Intake of free sugars, in grams per day and as % of energy, was positively associated with wealth status. The same pattern was observed for the consumption of sugar-sweetened beverages.

## 1. Introduction

There is increasing concern that intake of dietary sugars—particularly in the form of sugar-sweetened beverages–increases overall energy intake and may reduce the intake of foods containing more nutritionally adequate calories, leading to weight gain [1], dental caries [2] and cardiovascular disease [3]. It has been traditionally recommended to decrease the intake of added sugar [4,5], defined as sugars added to foods during processing or preparation. More recently, several health organizations have moved the focus towards monitoring the amount of free sugars instead of added sugars in the diet [6,7,8]. The main difference between added and free sugars is that fruit juices are included within the definition of free sugars.

Due to these recently developed recommendations, most food composition tables do not include information on free sugar content, and labels on pre-packaged foods lack such descriptive information. One notable exception is the United States, where added sugars are mandatory on the food labels, and are included in the US Department of Agriculture (USDA) Food Pattern Equivalent Database (FPED), allowing the estimate of their intakes in the US population, based on the National Health and Nutrition Survey (NHANES) data [9].

There is no standardized method to estimate the content of free sugars in foods, and free sugars cannot be distinguished from naturally occurring sugars with chemical analyses. Therefore, the estimates must rely on one of the following facts, or a combination thereof: (a) available categorization of foods in the database, usually available as assignment to food groups; (b) knowledge of the ingredients in a typical recipe; (c) information about the content of other nutrients, mainly total sugars and fiber. Our multi-step approach applies several imputation rules based on food groups, for which it is known a priori that they either contain no naturally occurring sugars (e.g., fish) or that they do not contain any free sugars (mainly whole fruits). In addition, and especially for mixed dishes, a predictive model is applied, based on the nutrient content of the foods.

A previously published paper developed a common-sense rule to estimate free sugars from added sugars using a food composition database from commercially available products [10]. However, this method was not validated against other databases.

In Louie et al. [6], a methodology to estimate the content of added sugars was developed and applied to the Australian Food Composition Table (FCT) and can be easily extended to free sugars. This 10-step procedure can, in principle, be applied to any FCT, but some of the steps require manual, time-consuming annotation and are very subjective. In fact, the reliability of the method was evaluated by comparing the estimates made by two researchers: for 20% of food items. The two researchers did not use the same steps, and for certain steps, agreement was below 50%. Although the authors concluded that this 10-step methodology can estimate added sugars content of foods with good reliability, it suggested that development of additional objective steps might rather improve the reliability of the method.

There is a knowledge gap around the consumption of free sugars in south-eastern Asian countries, due to the lack of appropriate food databases. The Philippines have adopted the WHO recommendations on free sugars in 2018 [11] and conduct a well-developed national nutrition survey to monitor the adherence of the Filipino population to the local dietary guidelines [12]. However, the information about free sugars is lacking in the FCT.

In this study, we propose an alternative method to estimate the content of added and free sugars in a FCT, requiring a minimal number of manual annotations and subjective steps. The method relies on availability of data on total sugars, food groups and nutrients readily available in FCTs (protein, carbohydrates, fiber, total fat, saturated fat and sodium). We applied our method to provide estimates of the intake of free sugars in the adult Filipino population based on the 2018 National Nutrition Survey (NNS). We then analyzed the association of these estimated intakes with wealth status and BMI.

## 2. Materials and Methods

### 2.1. Definition of Free Sugars

**Figure 1:**
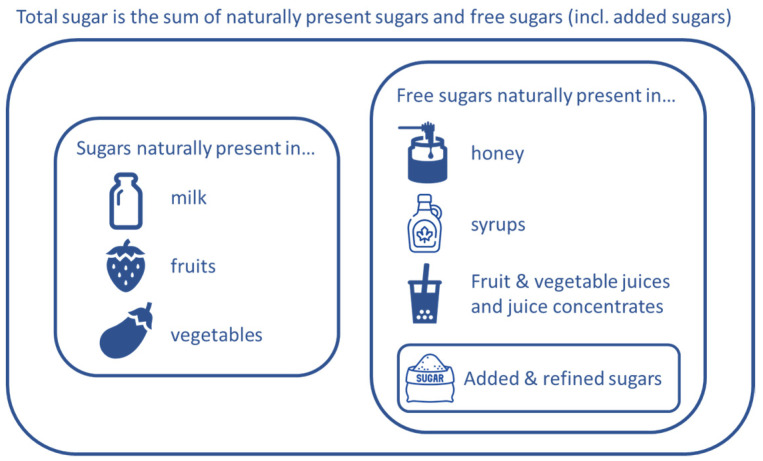
Schematic description of total sugars, free sugars and added sugars.

According to the European Food Safety Authority (EFSA), added sugars comprise all sugars which are added to food by the manufacturer, cook or consumer, such as glucose, fructose, sucrose, starch hydrolysates and other isolated sugar preparations [8]. Free sugars are defined, according to the WHO and the EFSA, as added sugars plus sugars naturally present in honey, syrups, fruit juices and fruit juice concentrates [4]. Both added and free sugars exclude the sugars that naturally occur in dairy products and intact fruit and vegetables. Refer to Figure 1.

### 2.2. Development of a Database of Free Sugars for the Philippines

Estimates of free sugar content were added to the electronic data files from the Philippines food composition database (PhilFCT) by adapting the method proposed by Louie et al. [6] Our method applies steps 1 to 3 of the 10-step methodology developed by Louie et al. [6] and replaces the remaining steps with an automatic data-driven estimation. The first three steps are based on objective criteria leaving less space for inter-researcher guesses.

All the steps rely on availability of data for total sugars (see Table 1). Steps 2 and 3 additionally rely on a categorization of the food items, that is usually available in FCTs in the form of food groups and subgroups (see Table 2). In the Philippines’ FCT, a 3-level categorization was available. For example, the item “Biscuit, wholemeal crackers” is categorized as Cereals and cereal products/Other cereal products/Cookies-biscuits. Finally, step 4 relies also on availability of nutrients usually available in FCTs (protein, carbohydrates, fiber, total fat, saturated fat and sodium).

**Figure 2:**
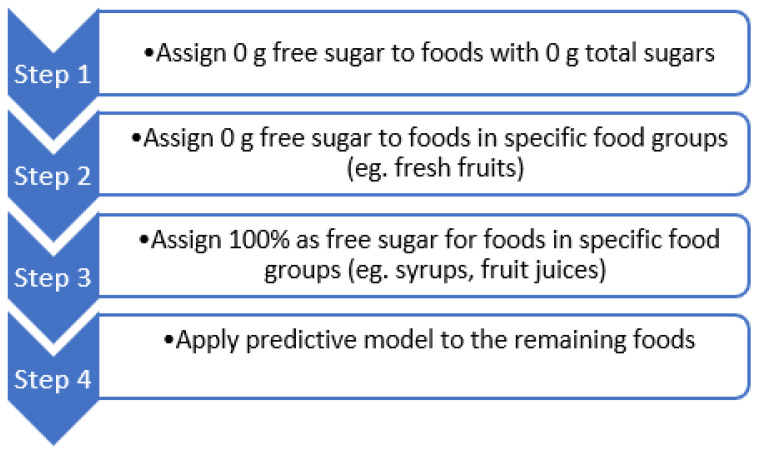
Step-by-step methodology of free sugars imputation.

The steps 1 to 4 used in our methodology are summarized in Figure 2 and described in what follows.

Step 1. Assign 0 g free sugar to foods with 0 g total sugars.
Step 2. Assign 0 g free sugar to foods in the following food groups: all spices, herbs, fats and oils; all plain cereal grains, pastas, rice and flours; eggs and egg products (except egg-based desserts); raw, fresh, dried, cooked foods (e.g., fruit, vegetables, legumes, meat, seafood) without addition of sugars; mixed dishes with no added sugar (decided based on ingredient information, e.g., recipe); non-sweetened beverages (e.g., coffees, tea, milks, alcoholic beverages); non-sugar-sweetened dairy products; nuts, coconut and seeds (except sweetened varieties and nut bars); plain breads and pastries without fillings (e.g., vanilla cream, chocolate).

These food groups were selected because they are either unprocessed or minimally processed with no added sugar.

Step 3. Assign 100% of total sugars as free sugar for foods in the following food groups: All non-dairy confectionery; breakfast cereals and cereal bars without fruits, chocolate, dairy or milk solids; coffee and beverage base with no milk solids, dry or made up with water; crumbed/battered meat and seafood; processed meats; sweetened beverages (e.g., soft drinks, sport drinks, flavored water); savory/sweet biscuits, cakes, donut and batter-based products without fruits, chocolate, or dairy products (decided based on ingredient information, e.g., recipe); soy beverages and soy yoghurt without added fruits; Sugar and syrups.

These food groups were selected as they do not contain sugars naturally, therefore, all the sugars present are likely to be free sugars.

Step 4. Apply predictive modeling to the remaining foods. We developed a stacked regression model [13], where each algorithm was tuned by 10-fold cross-validation. Stacking regressions is a method for forming linear combinations of different predictors to give improved prediction accuracy. We combined the predictions from:
  1. Support vector regression [14],
  2. Random forest [15],
  3. Extreme gradient boosted regression [16], and
  4. Rule fit regression [17].

Our strategy to train, test and validate the regression model was as follows.

To fit the model, we used:

- FNDDS 2013–2014 (US, 7618 foods)
- AUSNUT (Australia, 5740 foods)
- The model was then validated on a list of completely independent datasets:
- The Norwegian food composition table (1123 foods),
- The Danish food composition table (613 foods),
- 3082 recipes from the Internet, with complete information on the nutrients listed in Table 1, and on free sugars,
- 2 weekly menu plans, designed according to US dietary guidelines [18].

The reason to choose those countries was the availability of added or free sugars in their databases. Internet recipes were licensed from a commercial recipe database provider (Edamam LLC, New York, NY, USA) and contained additional ingredient mappings either to USDA SR28 or to the provider’s proprietary food composition table, for items that are not available in the USDA FCT, to provide detailed nutrition composition.

### 2.3. Estimating the Intake of Free Sugars

The Philippine National Nutrition Survey (NNS) is the official nationwide survey on nutritional status, diet and other lifestyle-related risk factors for noncommunicable diseases [12]. A 2-day, non-consecutive, 24 h food recall interview is conducted to estimate food intake. We used the first day of recall to estimate the intake of free sugars. We provide descriptive statistics of the intakes for the adult population, stratified by several socio-demographic factors (gender, age groups, BMI status, wealth status). BMI was adjusted for age for the group 4–18 years. Wealth status is a proxy measure of the long-term living standard of the household and was calculated by aggregating several components: household members’ educational backgrounds and occupations, type and tenure of housing unit, ownership of household assets, toilet facilities and garbage disposal systems, and source of drinking water, among others [19].

We analyzed the intake of free sugars as grams per day, and as percentage of daily caloric intake.

### 2.4. Statistical Analysis

We report the descriptive statistics of free sugar content (grams per 100 g) in the PhilFCT, overall and by food group.

We investigated the association of free sugar intakes with wealth status and with BMI status using a Kruskal–Wallis test followed by a post hoc Dunn test for pairwise comparison, with Benjamini–Hochberg correction for multiple testing. For subjects of age less than 19, the BMI status was adjusted for age.

Calculation of means, medians and standard error of continuous variables at daily level are weighted, using the survey weights (function svymean from the R package survey). Weighted general linear models were used to test for increasing trends between a continuous and an ordinal variable.

All calculations and analyses were performed in R, version 4.0.2.

## 3. Results

### 3.1. Development of a Database of Free Sugars

Although Louie et al. [6] consider step 1 to 6 as objectives, we decided to not apply their further steps, because the reliability decreases considerably from step 4. For this reason, aiming to decrease the number of manual annotations and possible inter-researcher errors, we used a different approach, and the remaining foods had their free sugars content estimated based on a regression model in which the information on nutrients is used.

More precisely, we developed a regression model taking as input seven nutrients: carbohydrate, fiber, protein, saturated fat, sodium, total fat, total sugar. These nutrients are usually well covered in most food databases (some examples are reported in Table 1).

A total of 1437 distinct foods were reported in the NNS, from a total of 1547 foods present in the database. There were 302 foods containing no sugars at all (Table 2), and 421 were imputed applying the data-driven model (Table 3). The remaining foods were imputed according to a-priori rules (steps 2 and 3), based on the food group.

The highest concentrations of free sugars were found in the syrups, cereals, and misc groups (Table 4); the group named “misc” includes the sugar-sweetened beverages as a subgroup.

### 3.2. Intakes

A total of 66,016 respondents had reported at least one day of intake, mostly in the age range 19–59 (49%, Table 5).

A total of 756,843 meals were reported in total, the most common ones being breakfast (29.7%), lunch (28.4%) and supper (27.1%). The mean daily intake of total sugars as reported was 28 (0.2) g/day (mean (SE)). Snack and breakfast were the meals with the highest content of free sugars. The daily intake of free sugars was estimated at 19 (0.1) g/day (mean (SE)). Measured as % of daily energy intake, this gave an overall average of 5% (0.03), with higher values for children (Table 6). Snacks and breakfast were the meals with the highest content of free sugars (Table 7).

BMI status was available for respondents aged 19 y or more (*n* = 40,099). Subjects in the obese and overweight groups had higher intakes of free sugars than subjects in the normal group (Dunn test, *p*-values < 0.01). When measured as % of energy, intakes were not significantly different between the groups. See Table 8. BMI adjusted for age z-scores (BAZ) were used for age below 19 y (Table 9). The difference between BAZ groups was not significant (Kruskal–Wallis, *p* = 0.87).

**Figure 3:**
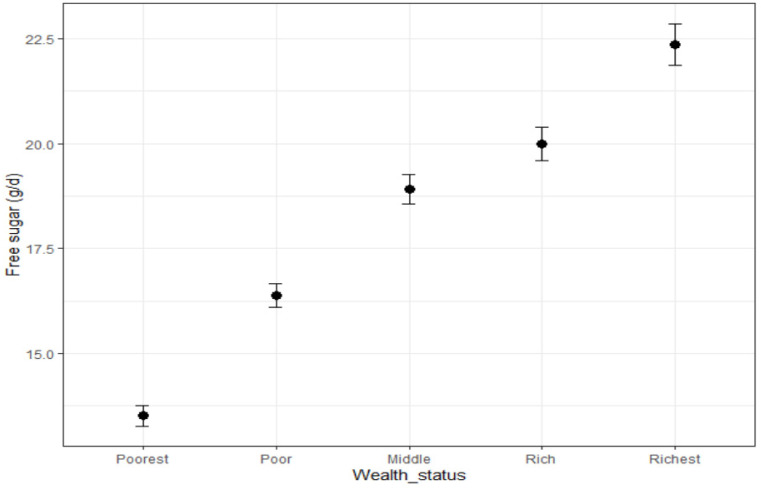
Intakes of free sugars by wealth status. Bars represent standard errors.

Wealth status was available for 65,678 respondents. The daily intake of free sugars was positively associated with wealth status, both when considered as amounts in grams per day, and as percentage of energy intake (Figure 3, Table 10 and Table 11). We also observed an increasing consumption of sugar-sweetened beverages with wealth status (Table 12); all *p*-values were significant (not shown).

## 4. Discussion

As free sugars have become a nutrient of public health concern, several diets and food quality indices/scores have free or added sugars as one of their components [17,18,19]. We developed a method to estimate the content of free sugars in food composition tables and applied it to the estimation of free sugar intakes in the Philippines. About 19.5% of the food had no sugars at all, 53.7% were imputed according to their assignment to specific food groups, and the remaining 26.8% were imputed using a data-driven approach, based on the content of carbohydrate, fiber, protein, saturated fat, sodium, total fat, total sugar. The data-driven method was applied to more than 60% of the cereal products and milk products, where total sugars can be partially coming from natural sources (e.g., milk or oats) and partially be added to the recipe. Correlations between predicted values and original values on the test datasets were very high, ranging from 0.89 to 0.96 (Table S1 Supplementary materials). The mean absolute error of the predictions ranged from 0.9 to 1.3 g/100 g (Table S1). We also evaluated the errors in g/day on 2 weekly menu plans, giving an estimate of how the errors combine when a multiplicity of foods is consumed in usual serving sizes (Table S1).

It is useful to compare our estimates with the intakes reported in other countries. In the US in 2017–2018, the average intake of added sugars was 17 teaspoons (71.4 g) for adults aged 20 and older [20], and 76 g for children 4–13 years old [21]. Intakes of free sugars, although not reported, should be expected to be comparable or higher. Our estimate for free sugars in the Philippines is much lower (19 g across all ages); however, this is true already for the intakes of total sugars, which were reported and not estimated (on average 28 g in the Philippines, against 107 g in the US) [22].

The 2009 Food Consumption Survey of Thai Population showed median intake of total sugar and sweeteners for all age groups ranging from 2.0 to 20.0 g per day among males and from 2.0 to 15.7 g per day among females, which is quite close to the average values observed for the Filipino population.

In general, it is known that consumption of sugar-sweetened beverages in the Asia-Pacific region is the lowest in the world [23].

Although estimated intakes were higher for overweight and obese, compared to normal BMI, these differences disappeared when intakes were converted to percent of caloric intake, similar to what was observed in the US population [20,21]. This is likely a result of selective under-reporting by overweight and obese individuals, namely of sugar-rich foods [20,22]. A strong association has been found between the preference for fat and energy-dense foods and obesity worldwide [22,23,24]. However, other studies showed no correlation between the preference for specific foods and the BMI status, whereas a recent study found evidence for energy-dense dietary pattern high in free sugars and saturated fatty acids (SFA) and low fiber and the obesity risk in Australian adults [25].

Estimated intakes of free sugars were positively associated with wealth status when measured in grams or as % of calories. This is opposite to what is observed in Western countries such as the US [24], where added sugars and foods with lower nutrient density are associated with lower socio-economic status. In January 2018, the Philippines began imposing a tax of 6 Philippine pesos per liter (around 13% of the cost of the product) on sweetened beverages to curb the obesity burden [25]. Conjecturally, this might induce poorest people to limit their consumption of such drinks, which is indeed what we observed in the data (Table 10, Figure 2). It has been reported that one month after implementation of the tax on 1 January 2018, prices of taxable sweetened beverages had increased by 16.6 to 20.6% and sales in sari-sari (convenience) stores declined by 8.7%.

## 5. Limitations

We acknowledge some limitations and areas of improvement in this work. We used a single 24 h recall, so our estimates may not be reflective of usual intakes. Our machine learning model was developed on Western data, and its applicability to Asian data might be not guaranteed. However, our database of internet recipes was multi-cultural, including many recipes from Asian countries. In addition, only less than 24% of the foods were fed into the model, the rest was processed during step 1 (11.6%), step 2 (53.4%), step 3 (11%). In addition, our model was not tailored for packaged products, in contrast with the work by Davies et al. Models for packaged products can exploit additional information from the label, particularly the list of ingredients, compensating for the fact that the relationships between nutrients can be altered in ultra-processed food.

## 6. Conclusions

We developed a method to estimate the content of free sugars in food composition tables, consisting of four objective steps and. Applied them to the estimation of free sugar intakes in the Philippines. A total of 19.5% of the foods had no sugars at all, 53.7% were imputed according to their assignment to specific food groups, and the remaining 26.8% were imputed using a data-driven approach, based on their nutritional content. The approach was validated on five independent datasets. Correlations between predicted values and original values on the test datasets were very high, ranging from 0.89 to 0.96 while the mean absolute error of the predictions ranged from 0.9 to 1.3 g/100 g. The daily intake of free sugars was estimated at 19.0 ± 0.1 g/day, corresponding to roughly 5% of daily energy intake. As expected, snacks and breakfast were the meals with the highest content of free sugars. Subjects in the obese and overweight groups had higher intakes of free sugars than subjects in the normal group. When measured as % of energy, intakes were not significantly different between the groups. Finally, the estimated intakes of free sugars were positively associated with wealth status, opposite to what is observed in western countries like the US.

## Figures and Tables

**Table 1:** Coverage of nutrients used in the regression model. SR = Standard Reference database from USDA; FNDDS: Food and Nutrient Database for Dietary Studies; AUSNUT = database used for the Australian nutrition survey.

FCT	Protein	Carbohydrates	Total Sugar	Fiber	Total Fat	Saturated Fat	Sodium
SR28 (US)	100.0%	100.0%	79.2%	93.2%	100.0%	96.0%	99.1%
FNDDS13-14 (US)	100.0%	100.0%	100.0%	100.0%	100.0%	100.0%	100.0%
AUSNUT (AU)	100.0%	100.0%	100.0%	100.0%	100.0%	100.0%	100.0%
PhilFCT (PH)	99.8%	99.9%	87.4%	91.8%	99.2%	80.1%	94.5%

**Table 2:** Number of foods imputed in each step.

Category	Imputation Step	*N*	%
alcoholic beverages	Assign 0 g of free sugars	10	0.6
eggs		18	1.2
fats and oils		28	1.8
fresh fish and meat		20	1.3
fresh meat		65	4.2
fruits		195	12.6
infant formula		5	0.3
organ meat		67	4.3
whole milk		3	0.2
vegetables		96	6.2
total sugar = 0		302	19.5
crackers		3	0.2
dried beans nuts and seeds		58	3.7
plain milk		4	0.3
plain cereals		49	3.2
spices		6	0.4
tubers		34	2.2
vegetables		2	0.1
vinegar		7	0.5
sugars and syrups	Assign 100% free sugar	54	3.5
coffee and beverage base with no milk		20	1.3
cookies		37	2.4
processed meat		40	2.6
soft drinks		3	0.2
All remaining foods	Predictive stacked regression model	415	26.8

**Table 3:** Number of foods imputed with the machine learning model, by food group.

	Cereals and Cereal Products	Fish Meat and Poultry	Milk and Milk Products	Misc	Other Fruits and Veg	Sugars and Syrups	Vit C Rich Foods
STEP							
Machine learning model	164 (63%)	30 (7%)	29 (66%)	69 (54%)	43(20%)	5 (8%)	6 (20%)

**Table 4:** Estimated content of free sugars, by food group, in grams per 100 g. The Misc group includes sugar-sweetened beverages, condiments and soups. The Vit C rich foods include citrus fruits, mangos, papayas and tomatoes. Free sugars in the ‘Other fruits and vegetables’ group come mainly from fruit juices.

	Free Sugars		Total Sugars	
	Mean (SD)	Median [Min, Max]	Mean (SD)	Median [Min, Max]
Cereals and cereal products (*n* = 258)	9.9 (12.2)	4.5 [0, 52.9]	13.4 (14.2)	6.75 [0, 65.9]
Dried beans nuts and seeds (*n* = 58)	0 (0)	0 [0, 0]	5.7 (5.8)	4.60 [0, 22.8]
Eggs (*n* = 18)	0 (0)	0 [0, 0]	0.7 (0.7)	0.5 [0, 2.5]
Fats and oils (*n* = 27)	0 (0)	0 [0, 0]	3.6 (7.6)	0 [0, 33.7]
Fish meat and poultry (*n* = 461)	0.3 (1.4)	0 [0, 20.2]	0.4 (1.5)	0 [0, 20.2]
Green leafy and yellow vegetables (*n* = 96)	0 (0)	0 [0, 0]	2.6 (3.6)	1.5 [0, 20.4]
Milk and milk products (*n* = 44)	3.9 (8.35)	0.38 [0, 43.7]	14.0 (17.6)	5.9 [0, 57.2]
Misc(*n* = 133)	10.9 (18.7)	1.40 [0, 78.0]	15.6 (23.4)	5.7 [0, 57.2]
Other fruits and veg (*n* = 215)	1.7 (5.71)	0 [0, 36.1]	8.35 (10.5)	4.5 [0, 67.6]
Starchy roots and tubers (*n* = 34)	0 (0)	0 [0, 0]	6.8 (9.2)	1.6 [0, 27.7]
Sugars and syrups (*n* = 62)	50.7 (27.9)	49.2 [0, 100]	51.7 (27.7)	50.2 [0, 100]
Vitamin C rich foods (*n* = 30)	0.0 (0.00)	0 [0, 0]	8.6 (5.7)	7.3 [0, 25.1]

**Table 5:** Description of the population.

	(*N* = 66,016)
**Age**	
Mean (SD)	32.0 (20.7)
Median [Min, Max]	28.6 [4.00, 98.0]
Sex	
Male	31,965 (48.4%)
Female	34,051 (51.6%)
**Household size**	
Mean (SD)	5.58 (2.54)
Median [Min, Max]	5.00 [1.00, 23.0]
**Wealth**	
Poorest	16,690 (25.3%)
Poor	15,954 (24.2%)
Middle	13,095 (19.8%)
Rich	10,790 (16.3%)
Richest	9149 (13.9%)
Missing	338 (0.5%)
**BMI**	
Mean (SD)	21.2 (5.17)
Median [Min, Max]	20.8 [6.82, 65.2]

**Table 6:** Free sugar intake as percent of daily energy, split by age group.

Age Group (Years)	Free Sugars % (SE)	Total Sugars % (SE)
**4–5** (*n* = 2007)	6.4 (0.2)	11.5 (0.3)
**6–9** (*n* = 8427)	5.6 (0.1)	8.5 (0.1)
**10–12** (*n* = 5768)	4.8 (0.1)	7.0 (0.1)
**13–15** (*n* = 5360)	4.7 (0.1)	6.6 (0.1)
**16–18** (*n* = 4355)	4.4 (0.1)	6.2 (0.1)
**19–49** (*n* = 24,682)	5.0 (0.0)	7.0 (0.0)
**50–59** (*n* = 7669)	5.1 (0.1)	7.7 (0.1)
**60–69** (*n* = 5012)	5.3 (0.1)	8.0 (0.1)
**70 and more** (*n* = 2736)	5.3 (0.1)	8.4 (0.2)

**Table 7:** Intakes of free sugars (g), by meal type. Mean, SE and median are un-weighted.

	Mean (SE)	Median [Min, Max]
**AM Snack** (*n* = 37,002)	4.6 (0.05)	0 [0, 251]
**Breakfast** (*n* = 224,842)	2.0 (0.01)	0 [0, 164]
**Late PM Snack** (*n* = 6316)	3.2 (0.09)	0 [0, 390]
**Lunch** (*n* = 215,052)	0.4 (0.01)	0 [0, 263]
**PM Snack** (*n* = 68,737)	4.9 (0.03)	0 [0, 390]
**Supper** (*n* = 204,894)	0.4 (0.01)	0 [0, 173]

**Table 8:** Intakes of free sugars by BMI status (age range 19+).

	Chronic Energy Deficiency (*n* = 3322)	Normal (*n* = 22,164)	Overweight (*n* = 10,784)	Obese (*n* = 3230)
**free sugar (% of kcal)**				
Mean (SE)	4.9 (0.6)	5.0 (0.0)	5.1 (0.1)	5.5 (0.1)
Median [Min, Max]	4.0 [0, 83]	3.8 [0, 40.5]	3.9 [0, 59]	4.2 [0, 33]
**free sugar (g/d)**				
Mean (SE)	20 (2.9)	20 (0.2)	21 (0.3)	21 (0.5)
Median [Min, Max]	15 [0, 284]	13 [0, 202]	14 [0, 228]	15 [0, 208]

**Table 9:** Intakes of free sugars by BAZ (BMI adjusted for age) status (age range 4–19).

	Severely Thin (*n* = 451)	Thin (*n* = 2132)	Normal (*n* = 20,949)	Overweight (*n* = 1587)	Obese (*n* = 879)
**free sugar (% of kcal)**					
Mean (SE)	4.9 (0.3)	5 (0.2)	5 (0.1)	5 (0.2)	5 (0.2)
Median [Min, Max]	3.4 [0, 83]	3.9 [0, 40.5]	3.7 [0, 59]	3.6 [0, 33]	3.9 [0, 30]
**free sugar (g/d)**					
Mean (SE)	17.1 (1.3)	16.7 (0.6)	17.3 (0.2)	20.5 (0.9)	23.4 (1.5)
Median [Min, Max]	11 [0, 284]	11 [0, 202]	11 [0, 228]	13 [0, 208]	16 [0, 190]

**Table 10:** Intakes of free sugars by wealth status.

	Poorest (*n* = 16,690)	Poor (*n* = 15,954)	Middle (*n* = 13,095)	Rich (*n* = 10,790)	Richest (*n* = 9149)
**Free sugars (% of kcal)**					
Mean (SE)	4 (0.2)	5 (0.0)	5 (0.1)	5 (0.1)	6 (0.1)
Median [Min, Max]	3 [0, 83]	4 [0, 56]	4 [0, 42]	4 [0, 59]	5 [0, 63]
**Free sugars (g/d)**					
Mean (SE)	14 (0.2)	17 (0.2)	20 (0.3)	21 (0.3)	24 (0.4)
Median [Min, Max]	10 [0, 284]	12 [0, 330]	14 [0, 302]	15 [0, 270]	17 [0, 418]

**Table 11:** Linear model for daily free sugar intake (g/d) regressed on the ordinal variable wealth. Estimates for the coefficients are in comparison with the “Poorest” level. Sample weights were used to fit the model. All *p*-values were <0.001.

	Free Sugars (Grams/Day)			Free Sugars (% of Energy)		
	Estimate	Std. Error	Pr (>|t|)	Estimate	Std. Error	Pr (>|t|)
**Intercept**	15.470	0.232	<2 × 10^−16^	4.174	0.059	<2 × 10^−16^
**Poor**	2.638	0.368	8.24 × 10^−13^	0.510	0.090	1.7 × 10^−8^
**Middle**	4.976	0.409	<2 × 10^−16^	0.980	0.094	<2 × 10^−16^
**Rich**	6.250	0.442	<2 × 10^−16^	1.207	0.102	<2 × 10^−16^
**Richest**	8.794	0.530	<2 × 10^−16^	1.757	0.116	<2 × 10^−16^

**Table 12:** Intakes of sugar-sweetened beverages by health status, adults 19 y and older.

	Poorest (*n* = 9247)	Poor (*n* = 9348)	Middle (*n* = 8038)	Rich (*n* = 6953)	Richest (*n* = 6327)
**SSB (servings per day)**					
Mean (SE)	0.2 (0.399)	0.2 (0.01)	0.3 (0.01)	0.3 (0.01)	0.4 (0.01)
Median [Min, Max]	0.0 [0, 5.29]	0.0 [0, 9.53]	0.1 [0, 6.34]	0.1 [0, 7.83]	0.1 [0, 8.46]

## Data Availability

The data used in this analysis were provided by the Filipino Nutrition Research Institute and is not publicly available.

## Supplementary Materials

The following supporting information can be downloaded at: https://www.mdpi.com/article/10.3390/nu15061343/s1, Figure S1. Missing values in the FCT; Table S1. Accuracy of the method on different datasets. In the first three lines, predictions are made on foods and recipes, and errors are evaluated in grams per 100 g. In the last two lines, we evaluated the errors in grams/day.

## Abbreviations

FCT	Food Composition Table
NNS	National Nutrition Survey
AUSNUT	Australian Food and Nutrient Database
SR28	Standard Reference version 28
PhilFCT	Philippines Food Composition Table
USDA	US Department of Agriculture
FPED	Food Pattern Equivalent Database
NHANES	National Health and Nutrition Survey
FNDDS	Food and Nutrient Database for Dietary Studies
BMI	Body mass Index
BAZ	BMI adjusted for age z-scores
RMSE	Root Mean Square Error
MAE	Mean Absolute Error
SFA	Saturated fatty acids
