# Supplementary material for: Estimation of Free Sugars in the Filipino Food Composition Table and Evaluation of Population-Level Intake
Source: Nutrients. 2023 Mar 9;15(6):1343. doi: 10.3390/nu15061343 (PMC10051834; doi:10.3390/nu15061343)
Supplement: Supplementary file 1 [file nutrients-15-01343-s001.zip › nutrients-2141046-supplementary.pdf]

nutrients

MDPI

Supplementary materials

Development and validation of the predictive model

The regression model takes as input variables the levels of Protein, total fat, saturated fat, carbohydrate, fiber, sugar and sodium. The choice of these nutrients was motivated mainly by their large coverage in food composition tables. However, missing values can still occur. In the Philippine's FCT, a majority of foods has complete information for these nutrients; however, a relatively small number of foods had missing values, especially for total sugar and saturated fat (Figure 4). Therefore, as a pre-processing step, we imputed the missing values using multiple imputation by chained equations [25].

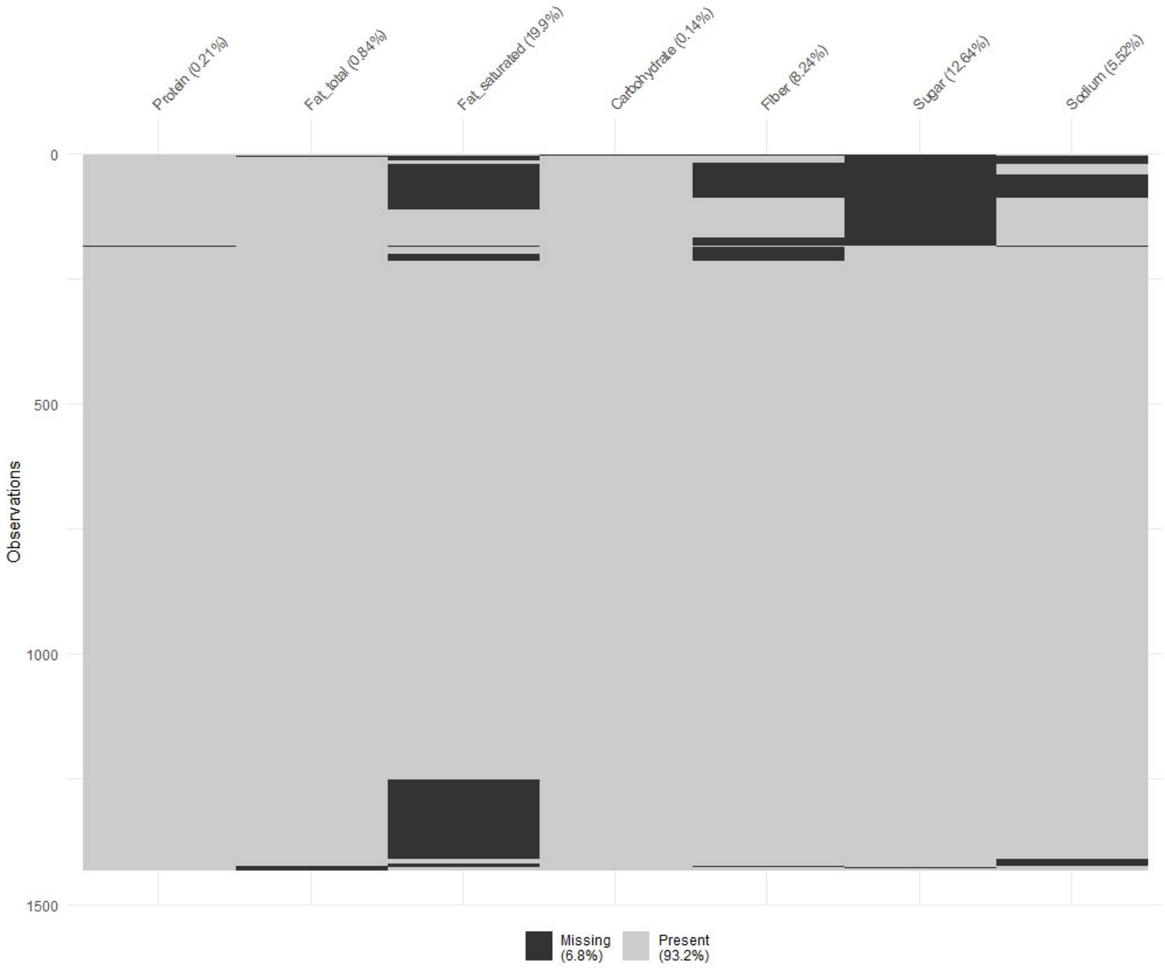

Figure S1. Missing values in the FCT.

For each validation dataset, we report three measures to evaluate the accuracy of the method (Table 13):

- R2: measures whether the estimated values and the actual values are linearly correlated
- RMSE: measures the average magnitude of the error, giving more importance to bigger errors
- MAE: measures the average magnitude of the error, less sensitive to outliers than RMSE

The model showed a good agreement between predicted and actual values on all the datasets.

Table S1. Accuracy of the method on different datasets. In the first three lines, predictions are made on foods and recipes, and errors are evaluated in grams per 100 grams. In the last two lines, we evaluated the errors in grams / day.

**Dataset**	**R2**	**RMSE**	**MAE**
Internet recipes	0.89	1.7	1
Norwegian FCT	0.96	2.8	1.3
Danish FCT	0.92	2.4	0.9
Menuplan-1	0.91	1.6	1.1
Menuplan-2	0.95	2.4	0.9
